# Supplementary figures and images for: ‘If relevant, yes; if not, no’: General practitioner (GP) users and GP perceptions about asking ethnicity questions in Irish general practice: A qualitative analysis using Normalization Process Theory
Source: PLoS One. 2021 May 12;16(5):e0251192. doi: 10.1371/journal.pone.0251192 (PMC8115799; doi:10.1371/journal.pone.0251192)

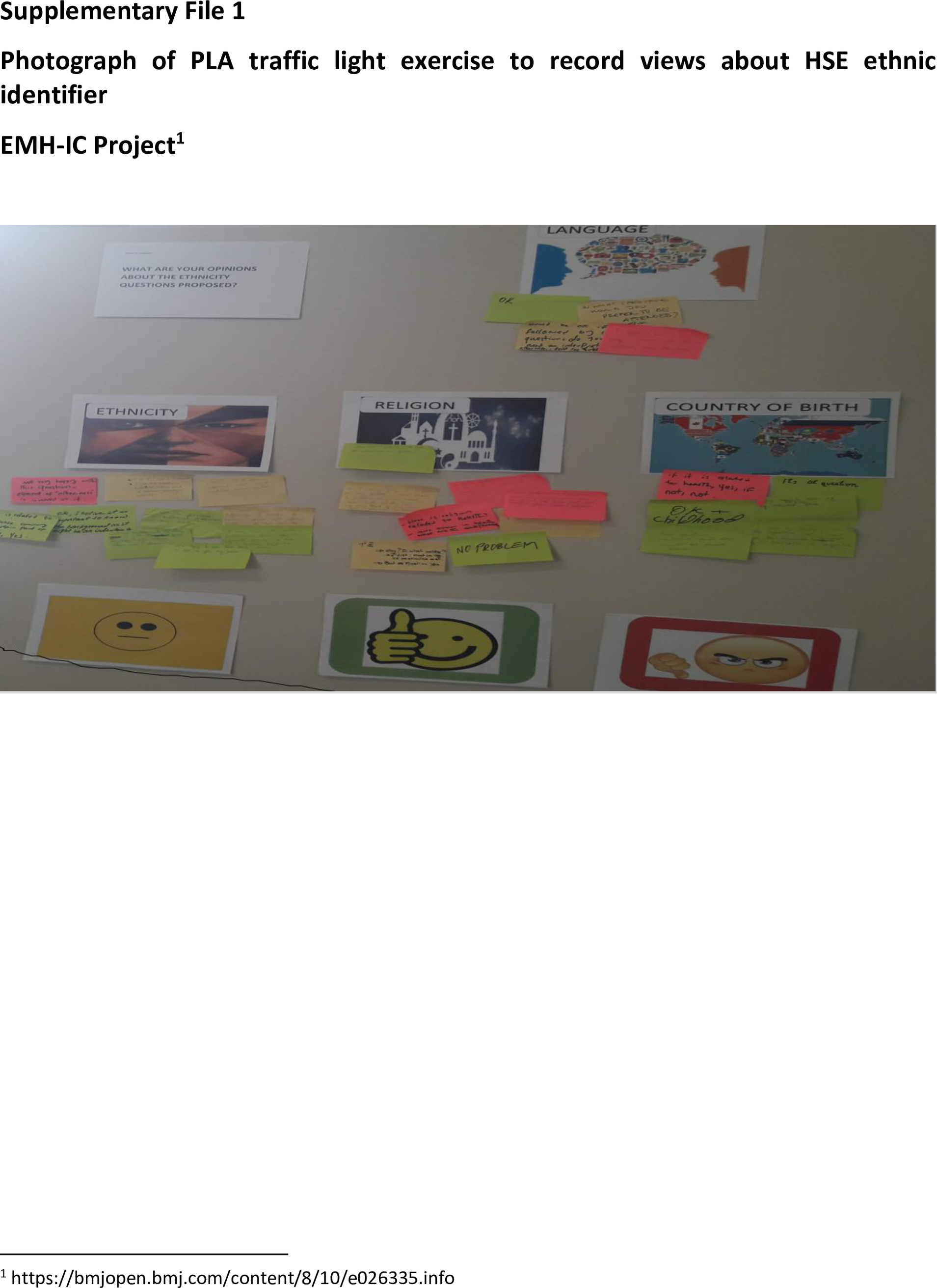

Supplement: S1 File — (TIF) [file pone.0251192.s001.tif]

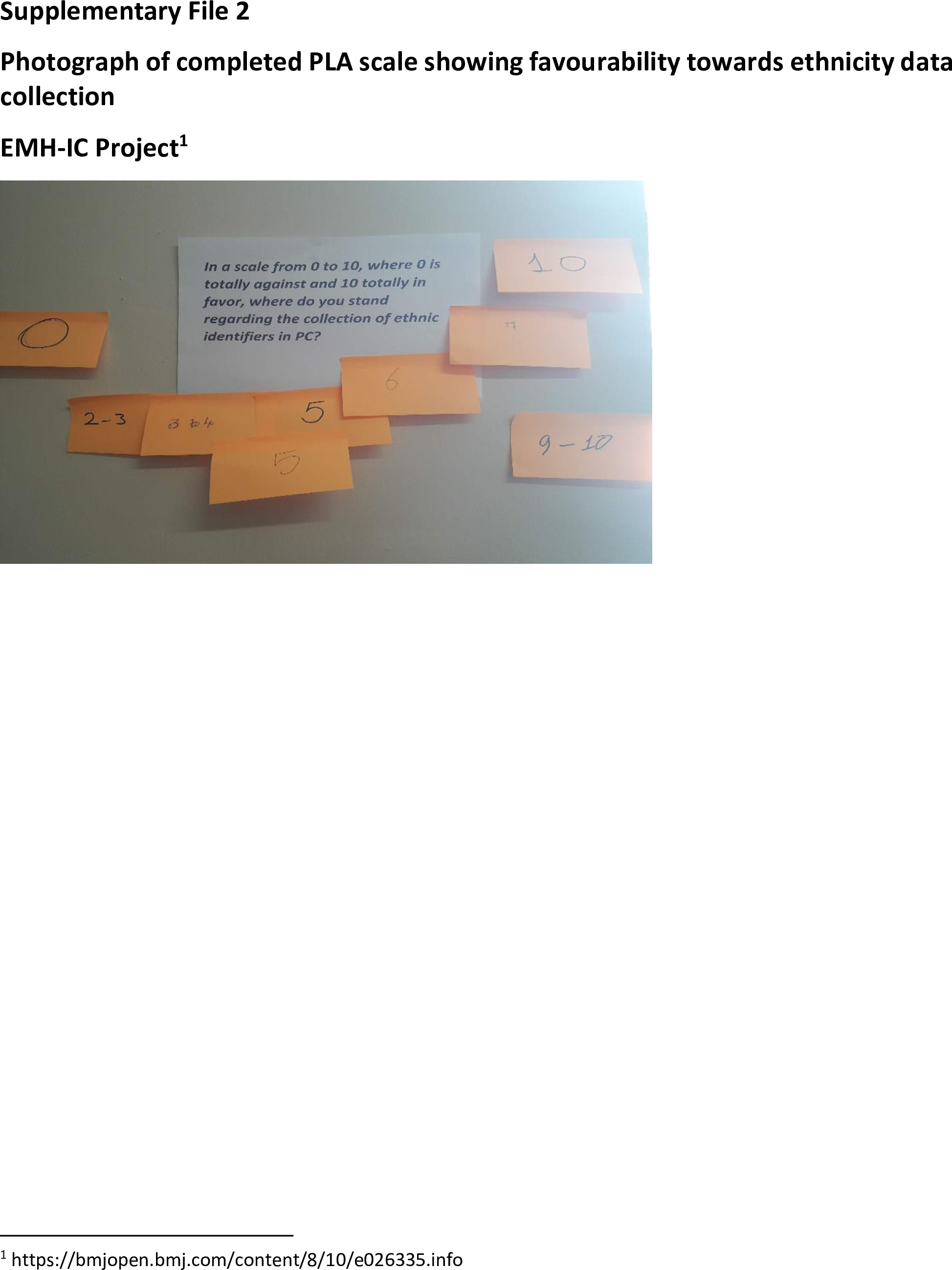

Supplement: S2 File — (TIF) [file pone.0251192.s002.tif]

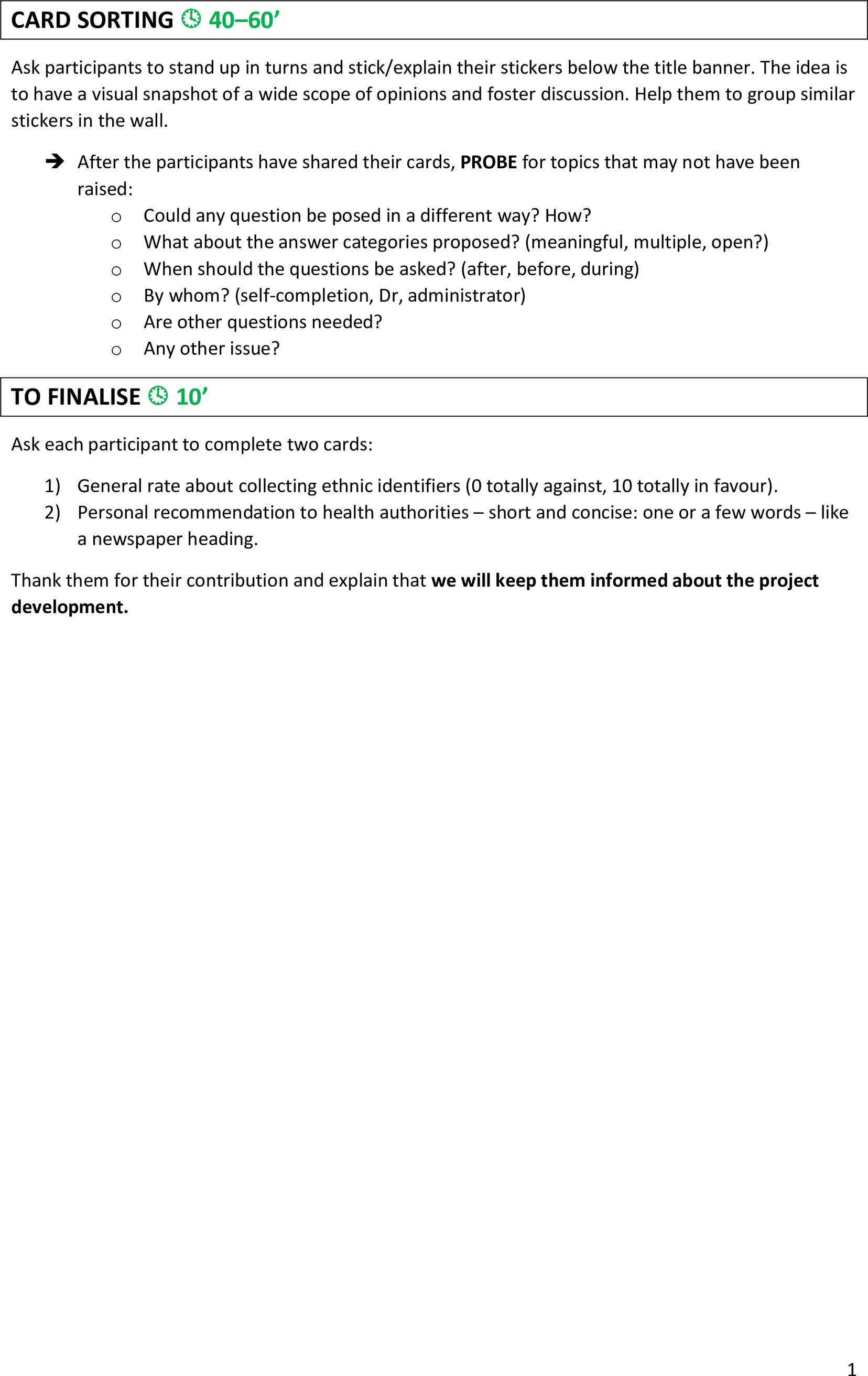

Supplement: S3 File — (TIF) [file pone.0251192.s003.tif]

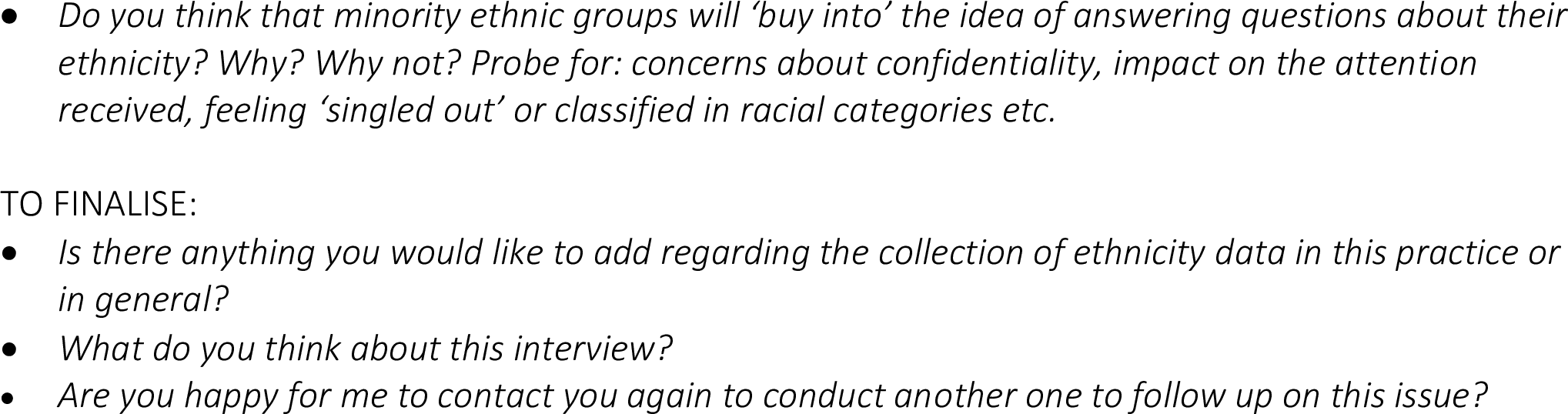

Supplement: S4 File — (TIF) [file pone.0251192.s004.tif]
